# Age, Period, and Cohort Effects in Alcohol Use in the United States in the 20th and 21st Centuries: Implications for the Coming Decades

**DOI:** 10.35946/arcr.v42.1.02

**Published:** 2022-01-13

**Authors:** Katherine M. Keyes

**Affiliations:** Department of Epidemiology, Mailman School of Public Health, Columbia University, New York, New York

**Keywords:** alcohol, age-period-cohort, cohort effects, gender, socioeconomic status

## Abstract

This article is part of a Festschrift commemorating the 50th anniversary of the National Institute on Alcohol Abuse and Alcoholism (NIAAA). Established in 1970, first as part of the National Institute of Mental Health and later as an independent institute of the National Institutes of Health, NIAAA today is the world’s largest funding agency for alcohol research. In addition to its own intramural research program, NIAAA supports the entire spectrum of innovative basic, translational, and clinical research to advance the diagnosis, prevention, and treatment of alcohol use disorder and alcohol-related problems. To celebrate the anniversary, NIAAA hosted a 2-day symposium, “Alcohol Across the Lifespan: 50 Years of Evidence-Based Diagnosis, Prevention, and Treatment Research,” devoted to key topics within the field of alcohol research. This article is based on Dr. Keyes’ presentation at the event. NIAAA Director George F. Koob, Ph.D., serves as editor of the Festschrift.

Alcohol consumption, including any alcohol use; patterns of high-risk use, including binge drinking; and alcohol use disorder (AUD) incidence and prevalence, differs substantially over time and by life stage. Variation also occurs across demographic groups, and such differences themselves vary across time and place. In the first quarter of the 21st century, changes in incidence and prevalence of alcohol use and alcohol-related health consequences have been accelerating. Understanding the magnitude and direction of these changes informs hypotheses regarding the reasons underlying alcohol consumption changes across time and development, including both long-term historical changes as well as abrupt shifts. It also permits determining the optimal focus of research and targets of services. Such surveillance is informed by science and statistical considerations of variation by age, period, and cohort effects.

Age-, period-, and cohort-effect estimation has proved to be an extraordinarily useful framework for organizing and interpreting data, uncovering patterns, and identifying causes of trends in incidence and prevalence of many health conditions and mortality over time. This article provides an overview of the conceptual basis of such effects as related to alcohol consumption, and reviews recent studies of age-period-cohort variation, especially regarding gender, social class, and specific beverage and drinking patterns.

## Age, Period, and Cohort Effects and Their Importance

### Age Effects

Age effects refer to the effects of a person’s age on their health. They may be caused by the accumulation of exposure or social experiences; critical and sensitive developmental windows; or immunological periods of vulnerability, such as infancy and end of life. Extensive evidence documents that alcohol use is most likely to begin during adolescence or young adulthood, peak during the transition to adulthood, and generally decrease thereafter.^1^,^2^ However, these age patterns are not static; in the United States, for example, the onset and peak of alcohol use has been shifting in recent decades to a later point in development.^3^ Because onset and persistence of alcohol use are in part social phenomena and are amenable to policy interventions (e.g., changes in minimum legal drinking age laws),^4^ the specific structure and magnitude of age effects are historically variable. However, the general patterns of onset early in adult maturation, and desistence during adulthood, have been largely stable over historical time.

### Period Effects

Period effects refer to changes in outcome that affect all individuals alive in a particular period—that is, a year or set of years. Reasons for period effects include changing environmental or social factors that affect incidence and persistence of certain behaviors or disorders, policy or law changes, or other environmental conditions that affect health. For alcohol use, numerous factors have been associated with substantial changes in consumption patterns, including major policy initiatives to restrict access to alcohol, such as U.S. Prohibition from 1920 to 1933, and broad economic factors, such as booms and recessions that affect spending on nonessential goods. The general social climate for heavy drinking has also changed over time as advocacy movements placed the dangers of heavy consumption into stark focus, followed by policies to increase criminal sanctions on impaired driving.^5^ However, as detailed below, such policy changes are not simply period effects because they often impact age groups differently; therefore, their effects may manifest as cohort effects.

### Cohort Effects

Against the backdrop of age and period effects, cohort effects have also proven to be powerfully predictive of a range of health behavior, including alcohol use. Cohort effects can perhaps be most efficiently conceptualized as age-by-period interactions.^6^ For example, a cohort effect would be apparent if historical change across time in a health behavior such as alcohol consumption resulted in increasing overall prevalence (i.e., a period effect), but the increase in prevalence is faster or slower for people in different age groups (i.e., an age by period interaction). Cohort effects can also be conceptualized as a unique rate of an outcome for individuals depending on birth year.^7^

Before reviewing the current literature on cohort effects in alcohol use, it is important to understand that cohort effects are powerfully predictive of many health outcomes, and critical to consider when evaluating trends. There are numerous historical examples of particular birth cohorts with increased rates of disease outcomes and mortality in the United States, including all-cause mortality,^8^,^9^ tuberculosis,^10^ peptic ulcer,^11^ lung cancer,^12^ and other diseases. More recently, the strong influence of generational cohort effects is apparent in the leading U.S. contributors to premature mortality, including obesity, hepatitis C, drug overdose, and suicide.^13^–^16^ Similarly, numerous studies in recent decades have found that alcohol use and health outcomes related to heavy consumption cluster by birth cohort, as well as have exhibited age and period effects at various points in history. Cohort effects have long been documented in substance use,^17^,^18^ including alcohol use and alcohol-related harms,^19^ as described in more detail below.

## Recent Alcohol Use Time Trends in the United States

**Figure 1:**
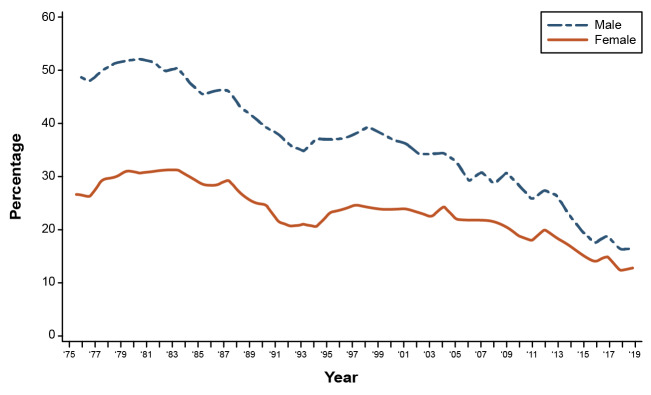
Trends in 2-week prevalence of binge drinking (≥5 or more drinks in about 2 hours), by gender *Source:* Adapted with permission from Johnston et al. (2019).^22^

Time trends in alcohol use and alcohol-related harms have been dynamic in the United States, especially over the last 2 decades. Among adolescents, the prevalence of alcohol use has declined. Data from two major nationally representative surveys—Monitoring the Future and the National Survey on Drug Use and Health—converge in demonstrating these reductions. Although the specific prevalence of any alcohol use and binge drinking differs between the two surveys, both document substantial, sustained reductions in adolescent drinking over the last 20 years.^20^,^21^ The most recently published data from the Monitoring the Future Study, depicted in Figure 1, show the trend in past 2-week binge drinking among 12th grade adolescents through 2019; as the figure shows, binge drinking declined from a peak in approximately 1982 to less than 20% for both boys and girls in 2019.^22^

**Figure 2:**
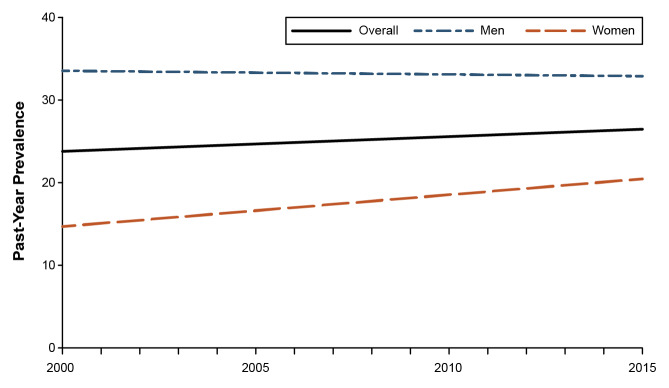
Simulated trend lines for past-year binge drinking prevalence overall and by gender Results are based on trend estimates from meta-analysis and use of 2002 NSDUH data to establish baseline prevalence. *Source:* Adapted with permission from Grucza et al.^23^

In contrast, adult alcohol use and binge drinking has been increasing. A meta-analysis of six national surveys of alcohol use found (Figure 2) that from 2000 to 2016, the overall prevalence of binge drinking increased approximately 7.5% per decade across the 2 decades analyzed.^23^ Importantly, however, these increases were primarily concentrated among women, as discussed further below.

The observation that changes over time in alcohol consumption differed by age immediately raises the possibility of cohort effects. Indeed, many studies using different data sources and analytical approaches have documented cohort effects for numerous alcohol-related outcomes. Generally, post-World War II U.S. birth cohorts had higher rates of consumption than earlier cohorts,^19^,^24^,^25^ driving much of the increase in consumption in the 1970s and 1980s. For many of these studies, however, reliance on retrospective recall is a common limitation. Avoiding this limitation, Kerr et al.^24^,^26^ used the National Alcohol Surveys, which reports current consumption patterns that are less subject to recall issues. These analyses documented that several birth cohorts had higher risks of alcohol consumption and binge drinking throughout the life course, especially men born in the late 1970s and women born in the early 1980s. In contrast, among cohorts born in the 1990s and later, alcohol use has consistently been declining during adolescence and early adulthood. However, those same cohorts have exhibited accelerating drinking after transition to adulthood.^27^

In sum, the cohorts of today’s adults who are now in their 30s and 40s were part of the historical shift toward declining alcohol consumption in adolescence. This decline is explained in part by shifts in the minimum legal drinking age across states, especially in the 1980s,^27^ yet declines continued thereafter, potentially aided by focused prevention efforts on reducing underage drinking. However, because drinking then accelerated during the transition to adulthood, adult rates of drinking did not benefit from these prevention efforts. Indeed, Patrick et al. (2019) have documented an overarching historical shift in the age effect on binge drinking among recently born cohorts; thus, the peak age of binge drinking in 1996 to 2004 was 2 years later than it was in 1976 to 1985.^3^

In addition to these overall age, period, and cohort effects, additional variation across other levels of dynamic change have implications for prevention, policy, and causal etiology assessments. Three areas of variation that have received substantial attention are gender, socioeconomic status, and beverage type.

### Effects of Gender

Men consume more alcohol and are more likely to have AUD compared with women,^1^ but the gender gap has been closing for decades in the United States and elsewhere.^19^,^25^ However, the manner in which the gender gap is closing differs by birth cohort. Among today’s birth cohorts of adolescents (i.e., those born in and around the same year), the gender gap is closing because for more than 30 years, alcohol consumption and binge drinking have declined among both boys and girls, but the decline is faster for boys than girls (see Figure 1).^28^ Conversely, in adults, alcohol consumption and binge drinking have increased, especially in the past 10 years, and those increases have been greater for women than for men (see Figure 2).^23^ The recent increases in drinking among women reflect the high-risk cohorts identified by Kerr et al.^26^ as they age into middle-adulthood. Interestingly, compared to earlier generations, these cohorts of women progressed through adolescence with lower alcohol use and binge drinking, yet had a faster acceleration of their drinking during the transition to adulthood, resulting in high levels of alcohol use and strong cohort effects in adulthood.^27^

Additional analyses have indicated that the increases in alcohol consumption and binge drinking among women in midlife are concentrated among those with high levels of education,^29^ occupational prestige,^30^ and income,^29^ suggesting that traditional gender norms sanctioning alcohol consumption are shifting among women now occupying traditionally male statuses and spaces. The human costs of these increases in consumption are reflected in alcohol-related mortality rates. These rates have doubled between 1999 and 2016,^31^ with the largest increases observed among women and adults emerging into midlife, consistent with alcohol consumption trends.

### Effects of Socioeconomic Status

Historically, the role of socioeconomic status has been a critical axis for examining trends over time in alcohol consumption, as exemplified by the higher consumption rates in adult women, who are increasingly occupying high socioeconomic positions. Overall, individuals with a higher socioeconomic status are less likely to fully abstain from alcohol compared to those with a lower status.^32^ The relationship between socioeconomic status and binge drinking or AUD, however, is more mixed and depends on the socioeconomic indicator, population, and time period analyzed.^33^–^35^ Further, population distributions of socioeconomic status are an outcome of economic conditions (i.e., income and wealth are functions of times of economic expansions and recessions); therefore, trends in socioeconomic status, and who achieves and maintains high status positions, are important potential drivers of population trends.

Renewed attention to theories of the relationship between social class and health has been prompted by evidence that recent increases in U.S. mortality, including alcohol-related and other substance-related mortality, are concentrated among men with less than a high school education.^36^ However, these findings run counter to available data on heavy drinking birth cohorts. The birth cohorts identified by Case and Deaton^36^ are different than the birth cohorts emerging into adulthood in the 1970s and 1980s or those of college age in 2002 to 2012, suggesting that the dynamics of alcohol-related harm are likely to substantially change in the decades to come. Indeed, National Alcohol Survey data show that cohort trends in U.S. alcohol consumption are primarily driven by changes in education.^37^ As more recent cohorts have entered college at higher rates, drinking and binge drinking have become concentrated in these college-attending young adults. The alcohol consumption cohort effect of those born in the late 1970s and early 1980s is attributable largely to their high rates of college attendance. Conversely, however, there may be signs of emerging socioeconomic differences when considered across gender (more on gendered trends in alcohol consumption below). For example, from 2002 to 2012, binge drinking was largely stable among college-attending young adults, but slightly increased among non-college enrolled women (from 29% to 33%) while decreasing among non-college-enrolled men.^38^ Continued surveillance of the role of socioeconomic status within trends in alcohol consumption, and beyond education into other indicators, is warranted.

### Effects of Beverage Type

Another important area for research is variation in alcohol consumption dynamics by type of alcoholic beverage. Although all alcoholic beverages are carcinogenic, beverage types vary in ethanol concentration and potential for harm, as well as in their prevalence and popularity across demographic groups. A growing literature indicates that the types of alcoholic beverages that individuals in the United States are consuming are dynamic and may depend on cohort. Kerr et al. (2004)^39^ found that pre-1940s cohorts preferred spirits throughout the life course compared with later cohorts. In contrast, cohorts born in the 1940s through 1970s, especially men, tended to prefer beer, and wine has been gaining dominance in beverage preferences among younger cohorts. These changes may be related at least in part to marketing and sales efforts by the alcohol industry to increase profits. For example, the increase in wine consumption, which has been observed in alcohol sales surveillance,^40^ is commensurate with the increases in income and education in the United States, as wine is marketed as a prestige product and is often sold at high price points. Additional analyses have found that the alcohol content of beverages is increasing in the United States,^41^,^42^ portending potential further harm and greater rates of AUD.

The dynamics of cohort effects on beverage preferences are particularly salient for the role of alcohol policy and reduction of alcohol-related harms. Sales restrictions and alcohol taxes have a substantial, demonstrable overall impact on population-level consumption and alcohol-related harms,^43^ although this varies to some extent by age of consumer, level of consumption, and beverage type.^44^ For example, tax variations by beverage type can influence trends in the consumption of particular beverages. Spirit and wine consumption is typically most sensitive to price and tax policy changes,^45^ and although consumption of spirits has been increasing in the United States in recent years, there has been little change in tax and price regulations. This suggests that one driver of the increase in spirits consumption is that they are becoming effectively less expensive over time. Beer and wine are also regulated differently in many states; thus, changing dynamics in the popularity of each beverage have implications for how effective beverage-specific alcohol taxes are in reducing sales and, consequently, harm. Regulations related to alcohol sales and consumption that can respond to market changes in beverage preferences (e.g., increased taxes on wine and spirits that reflect their growing share of the alcohol market) may be an important lever for promoting public health in the coming decades.

## Differences in Drinking Patterns Among Cohorts

Taken together, the literature on age, period, and cohort effects in alcohol research indicates that different cohorts have different drinking patterns and that socioeconomic and demographic factors are critical to contextualizing the observed trends. Although it is possible to document time and cohort trends with the available data, understanding why alcohol consumption patterns are changing is more challenging.

Certainly, alcohol policies play a fundamental role in determining population-level patterns of consumption, and the way that policies target particular demographic groups (intentionally or unintentionally) creates opportunities for cohort effects to emerge. For example, the adoption of a minimum legal drinking age of 21 across states throughout the 1980s mediates a portion of the decline in alcohol consumption among U.S. adolescents since then.^27^ However, consumption has continued to decline for decades after the increase in drinking age, suggesting that additional factors, such as the public health investment in underage drinking prevention, provided further benefits. Numerous other policies have shifted and impacted population-level alcohol consumption since the U.S. Prohibition, including restrictions on where and when alcohol can be sold, state monopolies on sales, criminal penalties for hazardous use, and others.^46^,^47^ These policies likely have affected different age groups in different ways, depending on their developmental stage when exposed to newly restrictive or permissive alcohol policies.

Of course, alcohol policies are not the only determinant of alcohol consumption and, consequently, of age, period, and cohort effects. Substantial research has evaluated the impact of social norms and social roles, as well as community and societal norms and values on changes in alcohol use over time.^48^,^49^ Social values have an inherent role in the use of alcohol, and the acceptability of drinking and drunkenness within and across social groups at different times and different life stages is potentially a powerful factor influencing population-level consumption. For example, heavy consumption on college campuses, especially within social institutions such as Greek life,^50^ is often normative and expected, but norms and values around alcohol use swiftly change as young adults encounter the social norms of early adulthood.^51^ Moreover, these normative trajectories and patterns become variable as societal roles and values themselves change. For example, religious attendance and the importance of religion have long been a robust predictor of decreased alcohol consumption.^52^ However, the centrality of religion to U.S. adolescents and adults has been declining for more than a decade,^53^ and this decline explains a portion of the cohort effects in binge drinking among today’s adults.^54^ Monitoring these and other broader societal changes is critical to determining the influences that mediate shifts in alcohol consumption over time.

For example, the coming years will be critical to determining the effects of health knowledge regarding alcohol-related risks on population consumption. For decades, low levels of alcohol consumption were considered protective, especially for cardiovascular health.^55^ The evidence supporting this hypothesis, however, was subject to substantial confounding,^56^ and dissemination of the message of alcohol’s protective effects was well-funded by the alcohol industry, which had a clear financial incentive.^55^ Recently, studies using large administrative databases and quasi-experimental designs, such as Mendelian randomization, have called into question and refuted the idea that a moderate level of alcohol consumption benefits health.^57^,^58^ The extent to which public health messages shift to reflect this change in scientific consensus may be important in reducing population-level alcohol-related harms. These changes may further manifest as cohort effects, as the dissemination and implementation of health information and guidelines are likely to affect age groups differently as they progress through the life course.

## Conclusions

Alcohol consumption continues to be a leading contributor to morbidity and mortality, both in the United States and worldwide. Although significant progress in reducing adolescent and young adult alcohol use has been achieved and sustained for decades, it is offset by increases in drinking during the transition to adulthood. The cohorts currently at midlife, especially women, are increasing alcohol consumption and binge drinking at greater levels than other cohorts, portending health consequences that may persist for decades. Understanding the motivations for consumption, destigmatizing the use of services to reduce consumption, and increasing the availability and accessibility of such services are necessary to improve population health. Moreover, age, period, and cohort effect estimations are critical surveillance tools for epidemiology and population health research. Such assessments have already answered critical questions and uncovered patterns in the data that specifically identify high-risk groups requiring prevention and intervention efforts.
